# Accounting for changes in flood control delivered by ecosystems at the EU level

**DOI:** 10.1016/j.ecoser.2020.101142

**Published:** 2020-08

**Authors:** Sara Vallecillo, Georgia Kakoulaki, Alessandra La Notte, Luc Feyen, Francesco Dottori, Joachim Maes

**Affiliations:** European Commission – Joint Research Centre, Ispra, Italy

**Keywords:** Actual flow, Service providing areas, Service demanding areas, Accounting tables, Monetary valuation, Avoided damage cost

## Abstract

Ecosystem service accounts require quantifying the contribution of ecosystems to the society. However, estimation of the ecosystem service used (actual flow) remains still very challenging for regulating services. We developed an experimental ecosystem service account for flood control delivered by ecosystems including: 1) Biophysical modelling of ecosystem service potential, demand and actual flow. 2) Translation of the actual flow in monetary terms. 3) Compilation of accounting tables. Ultimately, we analysed changes in flood control between 2006 and 2012.

The value of flood control delivered by ecosystems in 2012 is estimated at about 16 billion euro. This value increased by 1.14% between 2006 and 2012. This increase is mainly due to the sprawl of artificial areas into floodplains that benefit from flood control delivered by ecosystems. However, the role of natural capital to control floods is slightly decreasing. This is confirmed by the increase of artificial areas not protected by ecosystems (+1.9%, unmet demand). The role of natural capital to control floods could be enhanced by restoring ecosystems upstream from this unmet demand and increase the ecosystems contribution to human well-being. The methodology makes a significant contribution to the assessment of ecosystem services flow and the accounting framework.

## Highlights

• We present a novel method to account for flood control as ecosystem service.
• Flood control includes different components: potential, demand and actual flow.
• We provide an experimental account in line with up-to-date integrated accounting systems.
• The estimated value of flood control at the EU level is about 16 billion euro.
• Artificial areas not protected by ecosystems increased between 2006 and 2012.

## 1 Introduction

Ecosystems provide benefits to people in a direct or indirect way contributing to human well-being; which is known as ecosystem services (ES). The amount of services that ecosystems can provide to people depends on complex interactions between ecosystem and socioeconomic systems. When assessing ES, it is possible to quantify three different components that are essential to understand and properly assess the amount of service used, and therefore, the benefit generated (Villamagna et al., 2013). These key components are: 1) ES potential, which is the amount of ES that can be delivered in a sustainable way; 2) ES demand which is the need for a specific ES by society; and 3) ES use or actual flow which is the amount of service that is mobilized (used) in a specific place and time (Burkhard and Maes, 2017, Villamagna et al., 2013). ES potential is usually mapped based on the ecosystem’s properties and conditions relevant to the service considered (Syrbe et al., 2017). Mapping ES demand depends on whether it is understood as risk reduction, preferences and values, direct use or consumption of goods and services (see Wolff et al. (2015) for further details). Comparisons between ES potential and demand are common in the literature (Nedkov and Burkhard, 2012, Schulp et al., 2014b, Stürck et al., 2014) providing useful results for policy support and land planning. However, these comparisons do not provide information on actual ES flow. Methods for a consistent quantification of actual ES flow are still under debate, especially for regulating ES (de Groot et al., 2010, Serna-Chavez et al., 2014, Sutherland et al., 2018, Villamagna et al., 2013). Ultimately, the amount of ES used depends on the spatial relationship between the ES potential and ES demand, which are usually more complex than a simple overlap (Costanza, 2008, Fisher et al., 2009, Syrbe and Walz, 2012).

Several examples exist on the assessment of actual ES flow as a function of the ES potential, ES demand and their spatial relationship. Baró et al. (2016) quantifies the ES use of nature-based recreation and air purification by integrating ES potential (or capacity) and ES demand. Similarly, water purification by ecosystems is estimated as a function of the demand, where, similarly to air purification, the concentration of certain pollutants in the environment is used as a proxy to estimate the amount of ES that is actually needed (La Notte et al., 2015). The use of crop pollination can also increase when more pollination-dependent crops are present (Lautenbach et al., 2012). However, the same spatial framework is not applied on other services such as soil erosion and flood control, in which the actual ES flow is quantified without integrating the demand and/or beneficiaries (Barbedo et al., 2014, Grizzetti et al., 2017, Guerra et al., 2016). This ultimately contradicts the notion of ecosystem services (Maes et al., 2013). More concretely, flood control by ecosystems can be assessed using hydrological models quantifying the reduction of the flood peak discharges (Grizzetti et al., 2017, Smithers et al., 2016). Nonetheless, the ecosystem’s function and processes regulating water flows become ES only when there is demand for it (Fisher et al., 2009). Actually, placing more beneficiaries across the landscape may have the effect of increasing service flows (Bagstad et al., 2014). Other studies on flood control (Nedkov and Burkhard, 2012, Stürck et al., 2014) integrate the assessment of the demand for the service; however, estimates on the actual flow are not provided. More sophisticated studies, based on the Artificial Intelligence for Ecosystem Services (ARIES) platform (Villa et al., 2014), integrate the service demand and its spatial relationship with ES potential to quantify the service flow (Bagstad et al., 2014, Zank et al., 2016). However, they are generally applied at municipal and provincial level and the development of customised models in ARIES (and further new algorithms through its k.Lab technology) requires a high level of technical skill (Sharps et al., 2017).

The assessment of actual ES flows is required for natural capital accounts (UN et al., 2014a, UN et al., 2014b). Setting up ecosystem accounts is among the targets of the EU Biodiversity Strategy to 2020 and it is accompanied with a growing interest on the integration of natural capital accounting into policy decisions (Schaefer et al., 2015). Therefore, further research is needed to define common and coherent terminology and methods quantifying the actual flow of the ecosystem service, considered in accounting as a ‘transaction’ from ecosystems to socioeconomic systems.

In recent years, losses from floods have increased considerably, due to an increase of the economic activity in flood zones in combination with heavier rainfall in parts of Europe (European Environment Agency, 2016). Flood-related impacts are expected to worsen due to the ongoing socioeconomic and climate changes (Arnell and Gosling, 2016, Feyen et al., 2012). Therefore, understanding and properly assessing the actual use of ecosystems in controlling floods is becoming of particular importance. Flood control, as an ecosystem service, is defined as the regulation of water flows by ecosystems that mitigates or prevents potential damage to economic assets (i.e., infrastructure, agriculture) and human lives (modified from CICES V.5.1, Haines-Young and Potschin (2018)). All ecosystems but in particular forests, heath and shrublands, grasslands and wetlands reduce runoff by retaining water in the soil and aquifers and by slowing down the water flow. This prevents the rapid runoff of surface water, hereby lowering peak runoff, and thus reducing the detrimental effects from flooding on citizens, farmland, and infrastructure.

The main objective of this study is to develop a practical methodology for assessing ecosystem service flows at pan-European level and integrate them into an accounting system, using flood control by ecosystems as a case study. The methodology follows the approach described in the United Nations System of Environmental-Economic Accounting- Experimental Ecosystem Accounts (SEEA EEA) (UN, 2017, UN et al., 2014b). Moreover, practical applications as the one presented here are needed to further develop a standard for ecosystem service accounting. The experimental accounting approach developed here is based on spatially explicit models to assess different components of flood control by ecosystems (i.e., ES potential to reduce runoff, the demand by socioeconomic systems for protection against river floods and the actual flow (or use) of the ecosystem service). Subsequently, we economically valued the actual flow of flood control to fill in the accounting tables in monetary terms.

Since tracking changes over time is one of the main goals of Natural Capital Accounts (NCA), we also assessed changes in flood control by ecosystems for the years for which enough data for the assessment at the EU level were available.

The European Commission has encouraged interventions in flood mitigation that seek “to work with nature rather than against it”, recognizing that mitigating flooding effects through land use adaptation measures are “better environmental options” (Directorate-General for the Environment, 2011). Sustainable ecosystem management for disaster risk reduction such as flood mitigation is now recognised as a priority measure in the Sendai Framework for Disaster Risk Reduction (European Union, 2018). However, the role of ecosystems providing protection against floods to society is often overlooked or undermined and the methodology proposed to account for flood protection as ecosystem service may significantly contribute to give support to these policies.

## 2 Methods

For the mapping and assessment of the actual flow of flood control by ecosystems, we have adopted the ES framework that integrates the spatial dimension between the ES potential and the ES demand. The spatial relationship between these two components is perfectly accounted for with the spatially explicit mapping of the so-called Service Providing Areas (SPA) (Fisher et al., 2009, Sutherland et al., 2018, Syrbe and Walz, 2012) and Service Demanding Areas (SDA) (Orta Ortiz and Geneletti, 2018, Schirpke et al., 2019). By using the concept of SDA, we refer only to those areas in need for a given ES, but this does not necessarily imply that they benefit from ecosystems providing that service. Only when in SDA there is an effective ES flow, they can be considered as Service Benefiting Areas (SBA) in the sense of Syrbe and Grunewald (2017).

**Fig. 1:**
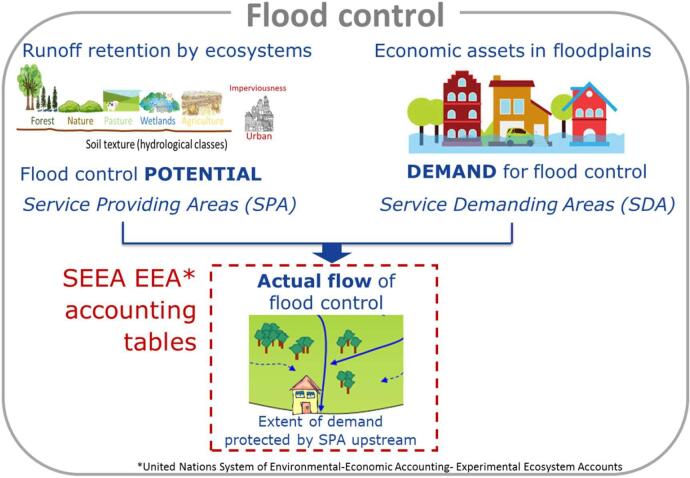
Scheme of the components assessed for flood control delivered by ecosystems.

The spatial relationship between SPA and SDA can be of different nature, depending on the ecological and socioeconomic process underlying the service (Costanza, 2008, Fisher et al., 2009, Syrbe and Walz, 2012). Flood control by ecosystems is directional-slope dependent, determined by the hydrogeological system following the slope of the terrain. It implies that the actual ES flow is only generated when the SDA lays downslope from the SPA and takes into account the whole river basin (Fig. 1).

Different spatial datasets were used to map the different components of flood control by ecosystems at the European Union (EU) level (Appendix A). The accounting layers of the CORINE land cover (CLC) map (EEA, 2017) were used as reference data defining the temporal and spatial resolution for the assessment of the difference components of flood control. The lack of imperviousness data for 2000 restricted the analysis into the years 2006 and 2012. For consistency, all input data were resampled to a common spatial resolution of 100 m. Maps of the different ES components were aggregated at sub-catchment level for visualization purposes. Sub-catchments are taken as spatial reference unit and are based on the Arc Hydro model, with an average sub-catchment size of 180 km^2^ (Bouraoui et al., 2009). Results are provided for the sub-catchments for which all datasets presented data. This excludes Malta, Cyprus and some areas of Croatia, Bulgaria and Finland. From here onwards, we refer to the study area as EU26. In this study, we focus only on river floods, which are the most frequent and most costly natural hazard (UNISDR, 2011).

The proposed methodology can be applied to any region or country whenever land cover and topographic data are available.

### 2.1 Ecosystem service potential

The mapping of the ES potential is the first step of the workflow for ecosystem services accounts. ES potential is also known as ‘supply’ or ‘capacity’ in the ecological literature (Maes et al., 2013, Villamagna et al., 2013); however, the use of these alternative terms would generate confusion within the accounting framework (UN et al., 2014b). An indicator of potential runoff retention was used to delineate the SPA for each sub-catchment, as suggested by Sutherland et al. (2018). The assessment of ES potential is based on five main steps described in the following section: 1) Curve number scoring for land cover classes; 2) Curve number adjustment by imperviousness; 3) Adjustment of the CN score by slope; 4) Integration of natural and semi-natural land cover in riparian zones, and 5) Mapping of SPA.

#### 2.1.1 Curve number scoring for land cover classes

The ecosystem contribution to reduce runoff by retaining water largely depends on the type of vegetation or land cover type, the soil hydraulic properties and the slope of the terrain. The Curve Number (CN) method, developed by the United States Department of Agriculture (2004), estimates runoff as a function of the land cover (or land use) and the soil type, for which a lookup table assigns different values. Soils show different hydrological soil properties depending on the textural classes present. The textural classes were reclassified into four categories following Zeng et al. (2017): A) Soils with low runoff potential (sand, loamy sand and sandy loam); B) Soils with moderate infiltration rates (silt, silt-loam and loam); C) Soils with slow infiltration rates (sandy clay-loam); and D) Soils with high runoff potential (clay, silty clay, silty clay-loam, sandy clay, clay-loam). Data of soil textural classes were provided by the European Soil Data Centre (ESDAC) (Ballabio et al., 2016, Joint Research Centre European Soil Data Centre (ESDAC), 2017).

The CN scores range from 0 to 100, with higher scores indicating higher runoff. Given the lack of empirical CN scores covering the EU, the role of each ecosystem type to control floods was initially quantified based on the correlation coefficients between the share of different land cover classes and the mean of ES provision obtained by different modelling techniques at the EU level (Schulp et al., 2014a). Correlation coefficients where rescaled between 0 and 100 (as the CN), and then this value was subtracted from 100 to derive reference CN with higher values in ecosystems generating higher runoff (Table 1). The reference CN scores shown in Table 1 were refined for the different CORINE land cover and soil categories (Appendix B explains the refinement of the CN scores).

**Table 1:** Reference values for the assessment of ecosystem potential to control floods.

Land cover type	Correlation coefficient [−1, 1]*	Correlation rescaled [0–100]	Reference Curve Number
Urban	−0.533	23	77
Pasture	0.055	53	47
Nature	0.283	64	36
Forest	0.609	80	20
Arable	−0.321	34	66

* From Schulp et al. (2014a).

#### 2.1.2 Curve number adjustment by imperviousness

Soil sealing or imperviousness is an ecosystem condition indicator (Maes et al., 2018) that reduces the natural capacity of soils to infiltrate water, driving therefore the ecosystem potential to control floods (United States Department of Agriculture, 1986). Soil sealing is not captured by the CLC map, considering all residential areas the same independently of their level of imperviousness, which may vary depending on the presence of vegetation (i.e., green roofs, parking areas with permeable surfaces). Thus we used imperviousness level (European Union, 2018) to refine the CN scores. Artificial areas are usually assigned a CN score of 98 (United States Department of Agriculture, 1986) that was corrected by imperviousness, according to Eq. (1):

(1) $${\mathrm{CN}}_{\mathrm{Total}}=98*\frac{\mathrm{Imp}}{100}+\left(1-\frac{\mathrm{Imp}}{100}\right)*{\mathrm{CN}}_{\mathrm{CLC}}$$

where *Imp* is the level of imperviousness (as percentage of impervious area within in each pixel at 100 m^2^) and ${\mathrm{CN}}_{\mathrm{CLC}}$ is the CN derived from the Table A.1 in Appendix B. When applying this model to other regions or countries, this step can be skipped if imperviousness data are not available.

#### 2.1.3 Adjustment of the curve number by slope

Since the CN method was initially developed for flat areas (slopes smaller than 5%), the effect of slope is not taken into account in the original CN method. Steeper slopes generate a faster movement of water within the landscape, reducing infiltration and therefore also the ecosystem contribution to control floods. As a consequence, a correction of the CN with respect to the slope was necessary by using Eq. (2) (Huang et al., 2006):

(2) $${\mathrm{CN}}_{\mathrm{Final}}={\mathrm{CN}}_{\mathrm{Total}}\frac{322.79+15.63(\alpha )}{\alpha +323.52}$$

where α is the slope ratio and ${\mathrm{CN}}_{\mathrm{Total}}$ is derived from Eq. (1). In this way, the ${\mathrm{CN}}_{\mathrm{Final}}$ combines as key variables land cover type, hydrological soil properties, imperviousness of the land surface and slope.

#### 2.1.4 Integration of natural and semi-natural land covers in riparian zones

Given the importance of natural and semi-natural land covers in riparian zones in controlling floods (Bennett and Simon, 2013), we assigned them the maximum ${\mathrm{CN}}_{\mathrm{Final}}$ score. Copernicus data provide a detailed map of riparian zones (European Union, 2018). As natural and semi-natural land covers, we considered agro-forestry areas [CLC 244], forest and semi-natural areas [CLC 311–313], scrub and/or herbaceous vegetation associations [CLC 321–324], wetlands [CLC 411–423].

The ${\mathrm{CN}}_{\mathrm{Final}}$ (higher scores corresponding to higher runoff) was transformed in a dimensionless indicator of potential runoff retention by subtracting to the maximum ${\mathrm{CN}}_{\mathrm{Final}}$ score of the reference year 2012 the CN score in a given location (i.e., complementary values of the${\mathrm{CN}}_{\mathrm{Final}})$. This way, high values indicate high potential of ecosystem to retain runoff.

#### 2.1.5 Mapping of service providing areas

The indicator of potential runoff retention provides spatially explicit data to identify key areas for flood control and to delineate SPA (i.e., when indicator is above a certain threshold). Using SPA instead of the indicator of potential runoff retention itself may be considered as an oversimplification, since a map with continuous data is converted into a Boolean map indicating presence or absence of SPA. Still, it is the basis for a spatial approach of ES at the landscape scale (Sutherland et al., 2018, Syrbe and Walz, 2012). Spatial assessments pairing SPA with the corresponding benefiting areas can provide insights into the role of spatial flows in the delivery of a particular ecosystem service (Serna-Chavez et al., 2014) as also demonstrated in previous examples of ecosystem service account (Vallecillo et al., 2019). Importantly, this conversion allows us also moving from a dimensionless indicator (potential runoff retention) to physical units to express ES as hectares of SPA per sub-catchment, which is preferred in an accounting context.

For the delineation of SPA, thresholds were set for three different coarse land cover groups. Setting the same threshold for the whole study areas would discard some relevant zones within cropland and urban areas playing a significant role in controlling floods for these types of ecosystems, which present distinct characteristics from semi-natural ecosystems. The three groups of land covers are: 1) artificial land covers; 2) agricultural land; and 3) the rest of land cover classes defined as natural or semi-natural land covers. The threshold for this last group was based on the average values for the different CLC classes of the mean ES potential for 2012 (used as reference year) minus the standard deviation (Appendix C). This criterion was not restrictive enough for agricultural and artificial land covers, given that the virtue of these land covers to control floods is intrinsically lower. In this case, we took the average values of the mean plus the standard deviation. For comparative purposes, the same thresholds calculated for the year 2012 were applied for 2006 to properly track changes over time.

Distinguishing different thresholds for each land cover group presents advantages from an ecosystem management point of view. For instance, SPA for semi-natural ecosystems excluded only 5% of their extent. The main land covers excluded as SPA are bare rocks and sparsely vegetated areas, which means that their role to control floods is low compared to other semi-natural ecosystems. Ecosystem restoration or nature-based solutions could be implemented to increase runoff retention in these land covers not considered as SPA. For agricultural areas, only 33% are considered SPA, including mainly agro-forestry areas, pastures, and areas with natural vegetation. Measures targeting the increase of natural vegetation in arable land for instance could increase the extent of SPA in agricultural areas. In the case of urban areas, 15% are SPA, which corresponds to artificial surfaces with low imperviousness level. Decrease of impervious areas (e.g., green roofs, parking areas with permeable surfaces) would increase runoff retention, acting therefore as SPA.

### 2.2 Ecosystem service demand

The service demanding areas (SDA) for flood control in this study are defined as the economic assets located in floodplains. For the mapping of the economic assets, we took artificial surfaces (Label 1 in CLC with grid code [111–142] and TeleAtlas roads) and agricultural areas (Label 1 CLC with grid code [211–244]) (Appendix D). As floodplains, we considered those defined by the flood hazard maps at the EU level for the maximum return period available, which is 500 years (Dottori et al., 2016). This map is available in JRC data catalogue (2018).

### 2.3 Ecosystem service use: the actual flow

The actual flow of flood control by ecosystems was only quantified for the areas in demand for flood control (SDA). For each 1 ha grid cell of SDA, we computed the share of the area upstream covered by SPA in the total upstream area $\mathrm{Ratio}\;{\mathrm{SPA}}_{\mathrm{up}}$ was then multiplied by the size of the grid cell to calculate the actual flow per grid cell of SDA (Eq. (3)):

(3) $$\mathrm{Actual}\;flow\;\left(ha\right)=\sum \left[Ratio\;{\mathrm{SPA}}_{\mathrm{up}}*{\mathrm{SDA}}_{\mathrm{Grid}\;cell\;size}\left(ha\right)\right]$$

where ‘${\mathrm{Ratio}\;SPA}_{\mathrm{up}}$’ is the ratio of the upstream area covered by SPA and 'Gridcellsize' refers to size of the pixel of demand.

The map of the actual ES flow of flood control is thus expressed as the number of hectares of demand (SDA) protected by upstream ecosystems (SPA) in a given year. The approach used in this report quantifies the role of the ecosystems to control floods in relative terms, compared to the best situation for flood control by ecosystems (i.e., when the entire upstream area of the demand is covered by SPA). This actual ES flow is thus dependent on changes in ecosystems situated upstream as well as on changes in the demand set by the economy (Fig. 1). Lastly, the actual flow per grid cell of SDA was summed up at sub-catchment level.

### 2.4 Unmet demand

The mapping of the actual ES flow as the number of hectares of demand protected by the ecosystem makes it feasible to map the unmet demand in the same terms. The unmet demand quantifies the part of the demand (economic assets) that is not covered by natural control by ecosystems. The unmet demand is quantified according to Eq. (4):

(4) $$\mathrm{Unmet}\;demand\;\left(ha\right)=Demand\left(ha\right)-Actual\;flow\left(ha\right)$$

An additional level of complexity which should be accounted for is that flooding areas usually contain artificial defence measures (e.g., levees, dykes) that are already in place guaranteeing certain level of protection. This should be considered when assessing the unmet demand. At the EU scale, data on the flood protection level are provided in terms of the return period of the flood event that can be borne by the defence measures already in place (European Commission – JRC, 2017). In the case of the Netherlands, the level of protection is high enough to safeguard all economic assets from flooding for the maximum return period considered (500 years). Therefore, we assumed that in this country, the demand for flood control is satisfied by the current level of protection and thus, the unmet demand was not calculated. The unmet demand was finally calculated as the percentage of the total demand for flood control at sub-catchment level (excluding the Netherlands).

It is important to highlight here that defence measures in place indirectly integrate the supporting role of ecosystems in controlling floods (Jongman et al., 2014). The protection level is designed to give protection up to a given flood return period taking into account a specific landscape setting (i.e., land covers). Changes in land cover upstream would alter water levels downstream and consequently the level of protection. It means that the presence of defence measures does not imply the lack of ecosystem’s role controlling floods, but rather ecosystems support the performance of defence measures. Actually, without the protective function of upstream ecosystems, more investment in artificial defence measures would be needed to maintain or guarantee the same level of protection. For this reason, the actual flow of flood control by ecosystems was quantified for the whole extent of the demand, including also the Netherlands, where protection level is the highest in the EU.

### 2.5 Monetary valuation

The actual ES flow of flood control quantified in biophysical terms is translated into monetary terms using avoided damage cost as valuation technique. This technique is consistent with the SEEA EEA Technical Recommendations (UN, 2017). This technique is based on exchange values and estimates the value of the damage that would occur if the ecosystem service were not present.

Estimation of the damage cost is adapted from the methodology presented in Feyen et al., 2012, Rojas et al., 2013. Damages are derived from depth-damage functions that express the damage cost in EUR/m^2^ as a function of the flood water depth (in meters) for different classes of land uses (i.e., buildings, commerce, industry, roads and agriculture). Damage functions for each class are adapted to the CLC classes identified as economic assets with demand for flood control following Huizinga (2007).

Damage functions vary among countries based on the Gross Domestic Product (GDP) per capita. No discounting or inflation rate is applied to the estimated values as they are calculated on the damage cost available.

In order to derive expected annual damage costs one should integrate damages for floods with different return periods to integrate the probabilistic terms of the different return periods. At EU level, data on water levels for different return periods are available in flood inundation maps (Appendix A) (Alfieri et al., 2015, Alfieri et al., 2014). The damage cost is calculated for the service demanding areas (SDA) applying the damage functions for the water depths of the available return periods at the EU level (i.e., 10, 20, 50, 100, 200, and 500 years).

The damage cost for each return period is then multiplied by the actual ES flow (Eq. (5)) as a proxy of the avoided cost (AC), required to estimate the monetary value of flood control by ecosystems. This proxy assumes that a higher damage is avoided if there are more upstream ecosystems contributing to control floods (actual ES flow).

(5) $$\mathrm{Avoided}\;cost\;\left(EUR\right)=Damage\left(EUR/m^{2}\right)\times Actual\;ES\;flow\left(m^{2}\right)$$

**Fig. 2:**
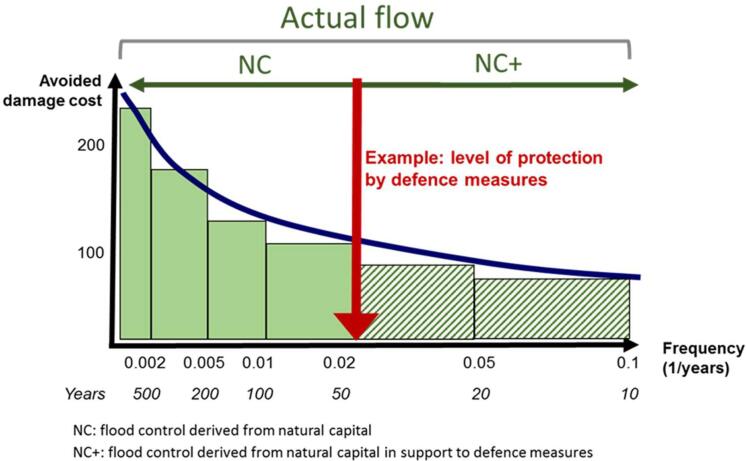
Illustrative example of actual flow in monetary terms and curve truncation.

The avoided cost estimated for each return period at grid cell level is then used to calculate the actual ES flow in monetary terms (Eq. (6), Fig. 2). This function is based on the equation used to estimate the Expected Annual Avoided Damage (Feyen et al., 2012).

(6) $$\mathrm{Actual}\;ES\;flow\;\left(EUR/year\right)=\sum_{10}^{500}\left(\left(f_{i}-f_{i-1}\right)*\frac{{\mathrm{AC}}_{i}+{\mathrm{AC}}_{i-1}}{2}\right)$$

where fi is the frequency of the return period (f = 1/return period i) and ${\mathrm{AC}}_{i}$ is the avoided cost (as calculated with Eq. (5)) estimated for the return period i.

However, in the monetary valuation, the role of artificial defence measures already in place is of especial relevance, since they reduce the damage generated by floods. Therefore, we also calculated the value of the actual flow considering the role of the defence measures by excluding in the estimates the potential damage of events with a return period lower than the protection level. The resulting value of the actual flow reflects the value of the ecosystem service where the only contribution to controlling floods is derived from natural capital (${\mathrm{Actual}\;flow}_{\mathrm{NC}}$). In this sense, Eq. (6) was truncated at the return period of the protection level (Fig. 2). For instance, if an area has a level of protection for a return period of 50 years, damage caused by return periods below this number will not be considered, decreasing accordingly the potential damage from floods (Eq. (7) is derived from the truncation of Eq. (6) for a return period of 50 years as an example):

(7) $$\mathrm{Actual}\;flow_{\mathrm{NC}}\;\left(EUR/Year\right)=\sum_{50}^{500}\left(\left(f_{i}-f_{i-1}\right)*\frac{{\mathrm{AC}}_{i}+{\mathrm{AC}}_{i-1}}{2}\right)$$

With this approach, we can also calculate the difference between the total value of the actual flow (Eq. (6)) and ${\mathrm{Actual}\;flow}_{\mathrm{NC}}$ (Eq. (7)) that would give the value of the actual flow of flood control when floods are controlled by both natural capital and defence measures (${\mathrm{Actual}\;flow}_{\mathrm{NC}+}$) (Fig. 2). As mentioned before, the presence of defence measures does not imply the lack of ecosystem’s role controlling floods, but rather ecosystems support the performance of defence measures.

### 2.6 Accounting tables

The core of ES accounts is focussed on the amount of ES used (the actual flow), which refers to the transaction between ecosystems and socio-economic systems (Fig. 1). The actual flow is reported in the both the supply and use tables, in biophysical and monetary terms (UN, 2017). While the supply table shows the contribution of different ecosystem types to generate the actual flow, the use table reports the contribution of the actual flow to the economic sectors and households using a given ES. Since both tables refer to the same ES flow, total values reported in the supply table are necessarily equal to total values of the use table, which is known as the ‘accounting identity’ (UN, 2017).

In the supply table, the actual flow is allocated to the different ecosystem types. For this allocation, we quantified first the extent of different ecosystem types shaping the SPA upstream from the demand in each country, since SPA are considered to generate the ES flow. Since the role of each ecosystem type controlling floods per unit area is highly variable (i.e., forest retain more runoff than cropland), the extent of each ecosystem type was weighted by a correction factor calculated with Eq. (8):

(8) $${\mathrm{Correction}\;factor}_{i}=\left(100-average\left({\mathrm{CN}}_{j\in i}\right)\right)/100$$

where i is the ecosystem type, and ${\mathrm{CN}}_{j\in i}$ is the CN of the land cover j belonging to the ecosystem type i (CN scores are shown in Appendix B). The ecosystems classification is based on Maes et al. (2013) (Appendix E shows their correspondence with CLC classes). The correction factors obtained are 0.27 for urban, 0.42 for cropland, 0.78 for woodland and forest, 0.56 for grassland, 0.64 for heathland, 0.33 sparsely vegetated land and 0.8 for wetland. The weighted extent (i.e., ecosystem extent in SPA multiplied by the correction factor) was then used to allocate the total actual flow in relative proportion to the values obtained.

In the case of the use table, the model output already provides the required information since land cover type and actual flow in monetary terms for each grid cell of demand are known. Correspondence between CLC classes with economic sectors and households defined by the Statistical Classification of Economic Activities in the European Community (NACE classification) is based on the description of each CLC class provided in Kosztra et al. (2017) (Appendix D).

## 3 Results

### 3.1 Biophysical maps of flood control

**Fig. 3:**
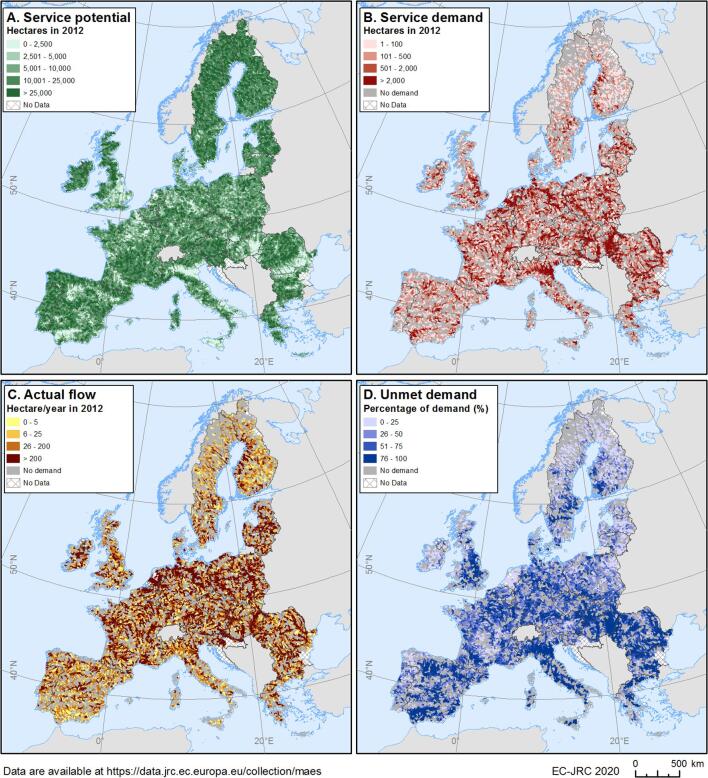
Flood control by ecosystems in 2012: A. Ecosystem service potential; B. Ecosystem service demand; C. Actual flow; and D. Unmet demand.

The maps with the different components of flood control by ecosystems are presented in Fig. 3, showing flood control potential (A); flood control demand (B); actual flow (C); and unmet demand for flood control (D).

In the EU26, the total service providing area (SPA) in 2012 represented 60% of the study area, showing higher flood control potential in forested areas in Europe and lower values in the main agricultural plains, e.g., in the east of the UK, southern Spain, the Po plain in Italy and in Romania. The total service demanding area (SDA) for flood control correspond to about 4% of the EU26 territory. ES demand is situated in river valleys, agricultural plains and in urban areas. The demand for flood control is composed by agricultural areas (88% of the total demand, with about 123,178 km^2^), while the remaining 12% is artificial land, with about 18,859 km^2^ in 2012 (Table 2). This extension of artificial areas in floodplains represents 10% of the total artificial area in the EU.

**Table 2:** Summary table at the EU level for the ecosystem service components for flood control.

Flood control at the EU level (EU26)				
	2006	2012	Changes	Changes (%)
**ES Potential (km^2^)**	**2,400,630**	**2,400,417**	**−213**	**−0.01%**
Gains (km^2^)			5,118	
Losses (km^2^)			5,331	
**ES Demand (km^2^)**	**142,270**	**142,037**	**−233**	**−0.16%**
By artificial areas (km^2^)	18,560	18,859	299	1.61%
By agricultural areas (km^2^)	123,709	123,178	−532	−0.43%
**ES Actual flow (km^2^)**	**41,880**	**41,696**	**−184**	**−0.44%**
In artificial areas (km^2^)	4,967	4,982	15	0.30%
In agricultural areas (km^2^)	36,913	36,714	−199	−0.54%
**Unmet demand (km^2^)**	**95,169**	**95,111**	**−58**	**−0.06%**
Unmet demand artificial areas (km^2^)	12,544	12,782	238	1.90%
Unmet demand agricultural areas (km^2^)	82,625	82,329	−296	−0.36%
**Monetary value actual flow (million euro)**	**16,127**	**16,312**	**185**	**1.14%**
In artificial areas (million euro)	15,323	15,512	189	1.23%
Value per unit of artificial demand (thousand EUR/km^2^)	826	823	−3	−0.37%
In agricultural areas (million euro)	804	799	−5	−0.58%
Value per unit of artificial demand (thousand EUR/km^2^)	6.5	6.5	0	−0.15%

The map of the actual flow shows darker colours when there is higher proportion of SPA upstream from the SDA, but also higher extent of demand benefiting from ecosystems controlling floods (Fig. 3C). The actual ES flow (or use) in 2012 is about 41,696 km^2^ (Table 2). This flow represents 29% of the service demand areas that are benefiting from ecosystems controlling floods. The unmet demand represent 67% of the total service demand area (the Netherlands is not included. See Section 2.4) and is mainly found in arable plains and large urban areas where the ES potential is generally low (Fig. 3D).

### 3.2 Compiled accounting tables

Table 3, Table 4 show an extract from the accounting tables in biophysical and monetary terms in the EU26. Accounting tables at country level are presented in Appendix F. In 2012, the value of flood control by ecosystems amounted to 16,312 million euro (Table 3). About 21% of this value is due to flood control derived from natural capital only (${\mathrm{Actual}\;flow}_{\mathrm{NC}}$). The remaining 79% represents the value of the service provided by ecosystems in support of artificial defence measures already in place (${\mathrm{Actual}\;flow}_{\mathrm{NC}+}$).

**Table 3:** Supply of the actual flow of flood control by ecosystem type at EU level (biophysical and monetary terms).

		Ecosystem types															
		Urban		Cropland		Grassland		Heathland and shrub		Woodland and forest		Sparsely vegetated land		Wetlands		Total	
Year 2006	Biophysical (km^2^)	262		3,159		7,727		724		29,329		2.5		677		41,880	
	Monetary (Million EUR)	89		1,012		3,099		350		11,244		0.9		332		16,127	
		NC	NC+	NC	NC+	NC	NC+	NC	NC+	NC	NC+	NC	NC+	NC	NC+	NC	NC+
		70	19	230	781	545	2,554	97	253	2,480	8,764	0.2	0.7	89	243	3,512	12,615
	Relative value* (EUR/km^2^)	416		628		6,089		1,925		7,049		15		3,383		3,779	

Year 2012	Biophysical (km^2^)	262		3,136		7,670		720		29,229		2.4		675		41,696	
	Monetary (Million EUR)	89		1,015		3,129		357		11,388		0.9		333		16,312	
		NC	NC+	NC	NC+	NC	NC+	NC	NC+	NC	NC+	NC	NC+	NC	NC+	NC	NC+
		71	19	233	782	548	2,581	100	256	2,506	8,883	0.2	0.7	89	244	3,547	12,765
	Relative value* (EUR/km^2^)	420		630		6,147		1,959		7,140		15		3,393		3,822	

* Relative value is calculated as the monetary value per ecosystem type divided by the extent of each specific ecosystem type.

**Table 4:** Use of the actual flow of flood control by economic units at EU level (biophysical and monetary terms).

		Economic units													
		Agriculture		Mining, manufacturing & energy production		Construction		Transport		Waste management		Other tertiary and Households		Total	
Year 2006	Biophysical (km^2^)	36,913		397		35		3,012		17		1,506		41,880	
	Monetary (Million EUR)	804		2,147		156		1,393		0.07		11,627		16,127	
		NC	NC+	NC	NC+	NC	NC+	NC	NC+	NC	NC+	NC	NC+	NC	NC+
		183	621	393	1,754	23	133	366	1,026	0.01	0.06	2,495	9,132	3,461	12,666

Year 2012	Biophysical (km^2^)	36,714		417		38		2,992		16		1,518		41,696	
	Monetary (Million EUR)	799		2,237		165		1,384		0.07		11,726		16,312	
		NC	NC+	NC	NC+	NC	NC+	NC	NC+	NC	NC+	NC	NC+	NC	NC+
		182	617	415	1,822	28	137	364	1,020	0.01	0.06	2,506	9,220	3,495	12,816

When looking at the different ecosystem types, around 70% of the total supply value is generated by woodland and forest, also showing a high value per square kilometre of ecosystem (Table 3). Also grasslands and wetlands revealed relatively high values per km^2^, while urban ecosystems and croplands showed the lowest values. Most of the supply in biophysical terms is used by agriculture (88% of the total) given the large extent of agricultural land in flooding areas (Table 4). However, the service flow in monetary terms is more valuable the tertiary sector and households (72% of the total), where ecosystems protect mainly residential buildings. This difference is due to a higher damage cost for residential areas than agricultural land, which differs by three orders of magnitude (e.g., in Belgium the maximum damage expected for residential area is about 718 EUR/m^2^ whereas for agricultural land it is about 0.73 EUR/m^2^).

### 3.3 Analysis of trends in flood control

Aggregated numbers at EU level show a decrease between 2006 and 2012 in the main components of flood control by ecosystems in biophysical terms: ES potential, ES demand, ES flow and the unmet demand (Table 2). On the contrary, in monetary terms the value of the actual flow of flood control has increased with 1.14% (Table 2). This increase is explained by the growth of artificial land benefiting from ecosystems protection (actual flow for artificial land increased by 0.3%), which is translated in an increase of the monetary value of 1.23%. Importantly, when looking at the value of the actual flow in relation to the amount of demand (EUR/km^2^) we see a decrease in the value of the ecosystem service for both artificial and agricultural land (by −0.37% and −0.15%, respectively). Although the percentage points of change appear relatively small, they still suggest that the protective role of the ecosystems is decreasing; especially for artificial areas where there is also a notable increase of the unmet demand (Table 2).

The ES potential in the EU26 did not show significant net changes between 2006 and 2012 (−0.01%). However, there is large spatial variability in the distribution of the SPA: with losses of SPA in the EU26 about 5330 km^2^ and gains of about 5118 km^2^ (Table 2). Changes in ES potential are mainly due to land-cover changes. Ecosystem extent accounts prove to be useful complementary information to provide a better understanding of the drivers of changes at country level (Ecosystem Extent Accounts for Europe currently undertaken by the EEA, work in progress). The approach adopted to model flood control also highlights the role of imperviousness as an important driver of change in the ES potential. Approximately 30% of the decrease of SPA in the EU26 is due to an increase in imperviousness.

At a larger degree than the ES potential, the demand for flood control has also decreased between 2006 and 2012 of about 0.16% (Table 2). Although this decrease might be considered negligible at EU level, more relevant changes are found when examining the demand for artificial and agricultural areas separately. The extent of artificial land demanding flood control has increased in all countries, showing in the EU26 an increase of 1.6%. On the contrary, the demand by agricultural land has decreased by 0.43% (Table 2).

As consequence of the decrease in ES potential and demand for flood control, the actual flow in biophysical terms has also decreased in the EU26 in a higher rate than the other two components (−0.44% between 2006 and 2012). The decrease in the actual flow highlights the importance of the spatial component defined by the directional flow between SPA and SDA, since it took place at a higher rate than the decrease in the demand (−0.16%), together with an insignificant decrease of the SPA (−0.01%). Ultimately, the impact of SPA changes will depend on the specific location where changes take place in relation to the demand areas.

Complementary to the changes in the actual flow, we also assessed changes in the unmet demand (Table 2). Changes in the total unmet demand show a decrease of −0.06% between 2006 and 2012, however the unmet demand notably increases for artificial areas (by +1.90%).

## 4 Discussion

This paper presents an experimental ecosystem account that quantifies the actual flow or use of flood control as ecosystem service based on the interaction between ecosystems and socio-economic systems. The model makes use of the best available data at EU level and it is also suitable for integration into an accounting system. The presented methodology makes a significant contribution to the ES assessment and accounting framework, highlighting the importance of assessing the different components of ES: ES potential, ES demand, actual ES flow and also the unmet demand for the ES. This assessment is spatially and temporally explicit to fully incorporate the key role of the spatial relationship between the ES potential and ES demand, and integrate the dynamic of changes over time, which ultimately will affect the benefit generated to the society. Such development is highly needed for regulating ES such as flood control; which are frequently undervalued when multiple ES are assessed given the complexity of measuring the benefits they generate (Sutherland et al., 2018). The proposed methodology can also be used to make forecast of flood control as ecosystem service under future land cover scenarios.

### 4.1 Flood control by ecosystems in the EU

In this experimental account, the monetary value of the actual flow of flood control by ecosystems in 2012 is estimated at about 16,312 million euro. The ecosystem with the largest contribution to flood control is forest, with a value of about 7 thousand EUR/km^2^ (Table 3). These values could be potentially used by economic and financial actors whose activities depend on the vulnerability of the territory they act on, e.g. investments on ecosystem restoration or on nature-based solutions could be seen as an opportunity for business by protecting economic assets and reduce therefore the damage generated by flooding.

Monetary values reported in this study are difficult to compare with those reported in the literature because of the different methodologies used. Smithers et al. (2016) estimated the value of forest in approximately 2.5 thousand EUR/km^2^, which is the same order of magnitude of the value reported in this study. Barth and Döll (2016) estimated the value of flood control for a riparian forest in about 430 thousand EUR/km^2^. They value only the role a riparian forest (i.e., key ecosystem in flood control) directly benefiting a city downstream, generating therefore a higher value than the reported in this study. Our approach valued the use of forest controlling floods for artificial areas and agricultural areas (with a lower damage cost than artificial surfaces) in relation to the total forest extent (that can be used or not).

The accounting framework we present here highlights the importance of expressing the value of flood control, not only in relation to the ecosystem extent providing the service (as described above), but also in relation to the extent of the demand areas. Actually, the value of the flood control by ecosystems varies significantly depending on the users of the ecosystem service. While the value of the ecosystem contribution to control floods in artificial areas is 823 thousand EUR per square kilometre of demand, for agricultural land is only 6.5 thousand EUR/km^2^ (Table 2).

The largest percentage of the total value of flood control by ecosystems (80%) takes place in support to artificial defence measures already in place (${\mathrm{Actual}\;flow}_{\mathrm{NC}+}$). Without the protective function of upstream ecosystems, more investments in artificial defence measures would be needed to maintain the same level of protection over time. The value of the ecosystem service where the only contribution to controlling floods is derived from natural capital is about 3.5 billion EUR (according to ${\mathrm{Actual}\;flow}_{\mathrm{NC}}$). This amount is about 55% of the total damage of floods estimated by Feyen et al. (2012) at about 6.4 billion EUR, where return periods protected by defence measures are discarded (i.e., similarly to the estimate of ${\mathrm{Actual}\;flow}_{\mathrm{NC}}$in our study). This may be understood as if the total flood damage in the absence of ecosystems would be at least 55% higher. However, the value of flood control provided in this study is to some extent underestimated since the damage curve used is based on simulated water levels reached for different return periods that already integrate the role of ecosystems (as represented by CLC 2006). Damages without ecosystem flood control would actually be much larger, since the water level reached for each return period would be also higher if the ecosystem was not there. Given that a situation without ecosystems cannot be realistically simulated, we use the damage function with ecosystems in place as a proxy for the avoided cost evaluation. This limitation could potentially be addressed by using simulations based on different ecosystem scenarios. However, this alternative method would be much more demanding in terms of data needs, technical skills to make the simulations of flooding areas and processing time, which makes it difficult to generate regular updates required for accounting.

The value of flood control increased between 2006 and 2012 (Table 2) mainly due to changes in the users. The sprawl of artificial areas in floodplains benefiting from ecosystems controlling floods increases the value of the ecosystem service. This situation shows that economic assets become more dependent on measures to protect them from floods, by ecosystems or artificial defence measures. Actually, in this case, the increase of the value of the actual ES flow should not be interpreted as a positive sign for natural capital. For this ecosystem service related to the reduction of occurrence probability of a flood event, the demand and especially the unmet demand are crucial. The increase of artificial areas in need for flood control (Table 2) is a consequence of poor spatial planning since urban sprawl is taking place in flooding areas putting at risk both economic assets and population. In addition, the increase of the unmet demand for artificial areas by 1.9% (Table 2) shows that there is a negative trend in the role of natural capital covering the need for flood control in these areas. This is also confirmed by the decrease of the monetary value for artificial areas in 3 thousand EUR/km^2^.

### 4.2 Contribution to ecosystem service accounting

The assessment of the actual ES flow for flood control as proposed in this study is consistent with the specifications defined in the SEEA EEA (UN et al., 2014b) and SEEA EEA Technical Recommendations (TR) (UN, 2017). Our approach allows building ecosystem services accounts in biophysical and monetary terms based on relatively low data requirements. The assessment of the actual ES flow modelled as a function of the ES potential and ES demand may benefit ES accounting by facilitating a direct link to the ecosystems reported in the supply table (through the ES potential) and to the economic units reported in the use table (through the ES demand). With this practical example, we have demonstrated the importance of assessing not only the actual flow of an ecosystem service, but also other relevant information such as the unmet demand; which allows a better understanding and appraisal of ecosystems services that act as buffers by mitigating the impact of flooding (see La Notte et al. (2019) for further discussion).

Comparison of the supply and use tables shows that urban and cropland ecosystems appear both as suppliers of flood control (in the supply table) and as users in the use table: agriculture (use table) corresponds to cropland (supply table), and the rest of economic units (use table) can be attributed to urban ecosystems (supply table) (Table 3, Table 4). This means that in urban and cropland ecosystems, vegetation and soils have the role of reducing runoff (although not at the same levels of forests, grassland or wetlands) while at the same time they are using the service for protection of their assets: artificial areas and agricultural areas (Appendix D). Therefore, effective management measures are especially encouraged for these ecosystem types, where an enhancement of the ES supply will return a direct benefit for the users. This is especially important for artificial surfaces, where the monetary value of the actual flow of the ecosystems service is higher, in absolute and relative terms (Table 2), and therefore management measures will be more beneficial. In situations in which suppliers (ecosystems) and users or beneficiaries (economic units) of ecosystem services spatially match or overlap, measures to enhance ecosystem condition are especially encouraged, given the difficulties of converting these land cover types to more natural ecosystems. A decrease in the level of imperviousness in artificial areas would enhance the ES potential rising therefore the use of the service and therefore the benefit to the society. These measures are especially encouraged in areas where there is an unmet demand by artificial surfaces. Although flood control accounts report useful information on the locations where measures are more urgently needed, local scale studies should be considered, together with the stakeholder involvement, for the final implementation of management measures in urban areas (Su et al., 2014).

The developed methodology is still in a testing phase of the SEEA EEA and should be interpreted in this context. Further development of this experimental account of flood control by ecosystems may consider calculating the actual ES flow weighting by the different values of potential runoff retention within each SPA (i.e., forest may retain more runoff than agricultural areas within the same SPA) and perform the corresponding sensitivity analysis. However, in this application, we discarded this option to be consistent with the approach used for the account of other ecosystem services (Vallecillo et al., 2018). Ultimately, the different role of each ecosystem type in providing the service was taken into account when compiling the supply table (see Section 2.6). Another important limitation is the potential bias in the selection of thresholds to delineate SPA, given the lack of scientific knowledge to set a realistic threshold. However, the values chosen as thresholds were suitable to track changes over time and make sound comparisons. Further development of the experimental account proposed here should include sensitivity analysis of the thresholds chosen.

In spite of the limitations, this method provides useful information to build flood control accounts in a consistent way and allows making comparisons over time. The role of precipitation has been implicitly included as a fix factor (climate related) over time in the modelling of floodplains (Dottori et al., 2016). We pose that annual precipitation data (meteorological) are not essential for flood control account, while they remain relevant for a service such as water supply. In fact, to the best of our knowledge, the only published accounts on flood control (Smithers et al., 2016) did not include precipitation data either. Modelling the actual flow of flood control based on annual precipitation data may result in an increase of ES flows when there is higher precipitation, even under circumstances in which the role of ecosystems controlling floods might be decreasing. This might lead to a misleading message since an increase in the actual ES flow might be interpreted as a positive fact from natural capital perspective. In addition, it might be masking important changes in the ecosystem contribution to control floods. We state that for ES accounts, changes in the actual ES flow should be explained by the drivers we consider in this study: ES potential, ES demand and their spatial relationship.

## 5 Conclusion

Flood control accounts are developed to provide policy support in relation to the mitigation of flood effects through sustainable ecosystem management. They may support the development of flood risk management plans integrating the role of ecosystems providing flood protection. Flood damage mitigation through nature-based solutions and ecosystem restoration is especially important under the expected increase of damage caused by river floods due to climate changes in the EU (Alfieri et al., 2018, Feyen et al., 2012). As pointed out before, this experimental ecosystem service account highlights the importance of managing ecosystem condition such as imperviousness in artificial surfaces when suppliers of the ecosystem service and users are the same. Ecosystem management measures (or nature-based solutions) should be prioritized in areas of unmet demand, especially in artificial surfaces, where the avoided damage cost by ecosystems is higher. The analysis of changes, even when we only considered a period of six years, raises awareness of the important role of flood control as ecosystem service. Although there is an increase of the demand for flood control, the role of ecosystems controlling floods is decreasing. These findings highlight the need of implementation of management actions to enhance ecosystem contribution to human well-being, as targeted in the 2020 EU Biodiversity Strategy.

## Declaration of Competing Interest

The authors declare that they have no known competing financial interests or personal relationships that could have appeared to influence the work reported in this paper

## Figures and Tables

**Table:** 

Input data	Source	Spatial resolution	Temporal coverage		
*Ecosystem service potential*					
Accounting layers CORINE land cover	https://sdi.eea.europa.eu/catalogue/srv/eng/catalog.search;jsessionid=ECE3C056F58790227AD6D6DCC72446D6#/home	100 m	2000	2006	2012
EU Dem 100 m > derive slope (m/m)	https://land.copernicus.eu/pan-european/satellite-derived-products/eu-dem/eu-dem-v1-0-and-derived-products/eu-dem-v1.0?tab=download	100 m	Static		
USDA soil textural classes: hydraulic properties	https://esdac.jrc.ec.europa.eu/resource-type/datasets	500 m	Static		
Imperviousness	https://land.copernicus.eu/pan-european/high-resolution-layers/imperviousness/view	100 m	NA^1^	2006	2012
Riparian zones	https://land.copernicus.eu/local/riparian-zones	Shapefile	Static		

*Ecosystem service demand*					
CORINE land cover: accounting layers > economic assets > agriculture and artificial	https://sdi.eea.europa.eu/catalogue/srv/eng/catalog.search;jsessionid=ECE3C056F58790227AD6D6DCC72446D6#/home	100 m	2000	2006	2012
Flood hazard map (return period 500 years)	https://data.jrc.ec.europa.eu/collection/id-0054	100 m	Static		
Road network	TeleAtlas	Shapefile-resampled at 100 m	Static		

*Actual flow (use)*					
EU Dem 100 m > flow direction and flow accumulation	https://land.copernicus.eu/pan-european/satellite-derived-products/eu-dem/eu-dem-v1-0-and-derived-products/eu-dem-v1.0?tab=download	100 m	Static		

*Monetary valuation*					
Estimated flood protection level (water depth)	https://data.jrc.ec.europa.eu/dataset/959355de-514a-4126-a969-27793cd775aa	5 km	Static		
Flood hazard map (all available return periods)	https://data.jrc.ec.europa.eu/collection/id-0054	100 m	Static		
Damage functions: Huizinga (2007)	https://link.springer.com/content/pdf/10.1007%2Fs10584-011–0339-7.pdf	Country	Static		
^1^NA: Not available					

**Table:** 

Coarse land cover classes^1^	Reference CN^1^	CORINE LC^2^	Refined CN	Comparison with Hong and Adler (2008)		Soil categories^4^			
				Average CN for all soil types^3^	Land cover types	A	B	C	D
Forest	20	Broad-leaved forest	**15**	**29**	Evergreen/deciduos broadleaf forest (mean)	8	15	18	19
		Coniferous forest	**25**	**29**	Evergreen/deciduos needleaf forest (mean)	14	24	30	32
		Mixed forest	**20**	**30**	Mixed forest	12	19	23	25

Nature	36	Transitional woodland-shrub	**28**	**34**	Closed shrubland	19	27	31	34
		Moors and heathland	**36**	**42**	Open shrubland	25	35	40	43
		Sclerophyllous vegetation	**36**	**42**	Open shrubland	25	35	40	43
		Bare rocks	**72**			64	73	74	77
		Sparsely vegetated areas	**63**	**63**	Barren or sparsely vegetated	56	64	65	68
		Burnt areas	**63**	**63**	Barren or sparsely vegetated	44	62	71	75
		Natural grassland	**41**	**42**	Grasslands	29	40	46	49

Pasture	47	Pasture	**47**	**42**	Grasslands	33	46	53	56

Arable	66	Non-irrigated arable land	**61**	**61**	Croplands	51	60	65	68
		Permanently irrigated land	**71**	**61**	Croplands	60	69	76	79
		Rice fields	**71**	**61**	Croplands	60	69	76	79

Urban	77	Artificial surfaces [except green urban areas]	**77**	**76**	Urban and built up	70	75	79	84
		Green urban areas^5^	36			24	35	41	43

Permanent crops	Not available	Vineyards	61	61	Croplands	49	57	65	72
		Fruit trees and berry plantations	61	61	Croplands	49	57	65	72
		Olive groves	61	61	Croplands	49	57	65	72

Heterogeneous agricultural areas	Not available	Annual crops associated with permanent crops	64			51	60	68	75
		Complex cultivation patterns	44	44	Cropland/natural vegetation mosaic	32	43	49	52
		Land principally occupied by agriculture, with significant areas of natural vegetation	44	44	Cropland/natural vegetation mosaic	32	43	49	52
		Agro-forestry areas	44	44	Cropland/natural vegetation mosaic	32	43	49	52

Wetlands	Not available	Inland marshes	20	20	Permanent wetlands	10	20	24	26
		Peat bogs	20	20	Permanent wetlands	10	20	24	26

**Table:** 

Land covers	CORINE Land Cover classes	Ecosystem service potential		Criteria	Value	Threshold
		Mean	Std. Dev.			
Artificial	Continuous urban fabric	10.59	5.28	Mean + Std.Dev	15.87	27
	Discontinuous urban fabric	20.55	6.25	Mean + Std.Dev	26.80	
	Industrial or commercial units	16.04	7.94	Mean + Std.Dev	23.98	
	Road and rail networks and associated land	19.95	6.80	Mean + Std.Dev	26.74	
	Port areas	12.68	8.25	Mean + Std.Dev	20.93	
	Airports	22.41	7.45	Mean + Std.Dev	29.86	
	Mineral extraction sites	25.52	5.03	Mean + Std.Dev	30.55	
	Dump sites	25.77	5.22	Mean + Std.Dev	30.99	
	Construction sites	21.81	6.79	Mean + Std.Dev	28.60	
	Sport and leisure facilities	25.92	5.01	Mean + Std.Dev	30.93	

Agricultural	Non-irrigated arable land	41.85	5.93	Mean + Std.Dev	47.78	52
	Permanently irrigated land	30.35	6.11	Mean + Std.Dev	36.46	
	Rice fields	30.75	5.23	Mean + Std.Dev	35.98	
	Vineyards	42.42	6.54	Mean + Std.Dev	48.97	
	Fruit trees and berry plantations	41.43	7.31	Mean + Std.Dev	48.74	
	Olive groves	39.10	7.65	Mean + Std.Dev	46.76	
	Pastures	56.70	6.76	Mean + Std.Dev	63.47	
	Annual crops associated with permanent crops	40.56	9.42	Mean + Std.Dev	49.98	
	Complex cultivation patterns	58.20	6.71	Mean + Std.Dev	64.91	
	Land principally occupied by agriculture	59.24	6.89	Mean + Std.Dev	66.13	
	Agro-forestry areas	61.33	5.45	Mean + Std.Dev	66.78	

Natural and semi-natural	Broad-leaved forest	87.05	3.57	Mean - Std.Dev	83.48	61
	Coniferous forest	84.04	5.46	Mean - Std.Dev	78.58	
	Mixed forest	85.51	4.43	Mean - Std.Dev	81.09	
	Natural grasslands	60.12	5.48	Mean - Std.Dev	54.63	
	Moors and heathland	68.93	5.65	Mean - Std.Dev	63.28	
	Sclerophyllous vegetation	65.80	4.08	Mean - Std.Dev	61.72	
	Transitional woodland-shrub	77.81	5.42	Mean - Std.Dev	72.39	
	Bare rocks	28.31	3.64	Mean - Std.Dev	24.67	
	Sparsely vegetated areas	38.43	3.99	Mean - Std.Dev	34.43	
	Burnt areas	40.23	10.08	Mean - Std.Dev	30.15	
	Inland marshes	82.64	6.49	Mean - Std.Dev	76.15	
	Peat bogs	87.79	5.25	Mean - Std.Dev	82.54	
	Green urban areas*	64.92	11.66	Mean - Std.Dev	53.26	
*They were considered in this group because of their different biophysical properties compared to the artificial and impervious land covers						

**Table:** 

Broad economic assets	CORINE Land Cover classes (LABEL 3)	Allocation to user of flood control^2^
Artificial surfaces	Continuous urban fabric	Other tertiary and households
	Discontinuous urban fabric	Other tertiary and households
	Green urban areas	Other tertiary and households
	Sport and leisure facilities	Other tertiary and households
	Road and rail networks and associated land (main roads from TeleAtlas are also added)	Transportation
	Port areas	Transportation
	Airports	Transportation
	Industrial or commercial units	Manufacturing and mining
	Mineral extraction sites	Manufacturing and mining
	Dump sites	Waste management
	Construction sites	Construction

Agricultural areas^1^	Non-irrigated arable land	Agriculture
	Permanently irrigated land	Agriculture
	Vineyards	Agriculture
	Fruit trees and berry plantations	Agriculture
	Olive groves	Agriculture
	Pastures	Agriculture
	Annual crops associated with permanent crops	Agriculture
	Complex cultivation patterns	Agriculture
	Land principally occupied by agriculture, with significant areas of natural vegetation	Agriculture
	Agro-forestry areas	Agriculture

**Table:** 

MAES ecosystem	CORINE Land Cover
Urban	Continuous urban fabric
	Discontinuous urban fabric
	Industrial or commercial units
	Road and rail networks and associated land
	Port areas
	Airports
	Mineral extraction sites
	Dump sites
	Construction sites
	Green urban areas
	Sport and leisure facilities
Cropland	Non-irrigated arable land
	Permanently irrigated land
	Rice fields
	Vineyards
	Fruit trees and berry plantations
	Olive groves
	Annual crops associated with permanent crops
	Complex cultivation patterns
	Land principally occupied by agriculture, with significant areas of natural vegetation
	Agro-forestry areas
Grassland	Natural grasslands
	Pastures
Heathland and shrub	Moors and heathland
	Sclerophyllous vegetation
Woodland and forest	Broad-leaved forest
	Coniferous forest
	Mixed forest
	Transitional woodland-shrub
Sparsely vegetated land	Beaches, dunes, sands
	Bare rocks
	Sparsely vegetated areas
	Burnt areas
	Glaciers and perpetual snow
Wetland	Inland marshes
	Peat bogs
Rivers and lakes	Water courses
	Water bodies
Marine inlets and transitional water	Salt marshes
	Salines
	Intertidal flats
	Coastal lagoons
	Estuaries

**Table:** 

Economic units			Ecosystem types														
	Economic sectors	Households	*Total*	Urban areas		Cropland		Grassland		Heathland and shrub		Woodland and forest		Sparsely vegetated land		Wetlands	
*Year 2006*				NC+	NC	NC+	NC	NC+	NC	NC+	NC	NC+	NC	NC+	NC	NC+	NC
*million euro*																	
AT			949	0.21	0.034	29.71	4.70	140.63	22.23	26.34	4.16	620.21	98.04	0.073	0.0115	2.68	0.42
BE			708	4.03	0.569	148.90	21.02	145.96	20.60	2.45	0.35	314.96	44.46	-	-	4.45	0.63
BG			67	0.02	0.012	4.48	2.21	4.52	2.23	0.29	0.15	35.22	17.39	0.003	0.0014	0.22	0.11
CZ			426	0.23	0.038	35.54	5.78	54.35	8.83	0.29	0.05	274.73	44.66	-	-	1.14	0.19
DE			3732	31.29	6.740	51.90	11.18	749.51	161.48	14.89	3.21	2203.58	474.75	0.020	0.0043	18.92	4.08
DK			22	0.46	0.157	4.09	1.39	0.60	0.20	0.36	0.12	10.03	3.40	-	-	0.55	0.19
EE			38	0.09	0.069	1.63	1.21	1.65	1.23	0.00	0.00	17.44	12.99	-	-	1.03	0.77
EL			36	0.00	0.002	2.04	1.93	1.79	1.69	2.44	2.31	12.07	11.42	0.012	0.0114	0.03	0.03
ES			478	0.12	0.077	28.18	17.99	37.29	23.81	54.00	34.47	171.85	109.69	0.040	0.0258	0.39	0.25
FI			804	0.83	0.324	19.12	7.48	0.20	0.08	3.94	1.54	523.99	205.03	0.001	0.0005	29.83	11.67
FR			2432	0.99	0.189	160.56	30.64	488.31	93.20	29.23	5.58	1354.50	258.52	0.221	0.0422	8.69	1.66
HR			54	0.00	0.005	0.21	9.53	0.06	2.56	0.00	0.12	0.91	40.56	0.0000	0.0002	0.00	0.13
HU			156	0.11	0.021	10.51	1.97	18.04	3.39	0.07	0.01	100.08	18.80	0.002	0.00028	2.15	0.40
IE			155	0.03	0.011	6.01	2.21	74.87	27.54	0.54	0.20	14.26	5.25	0.001	0.00029	17.54	6.45
IT			501	0.12	0.021	32.84	5.84	27.92	4.96	11.73	2.08	351.57	62.50	0.323	0.0574	0.88	0.16
LT			190	1.15	0.868	24.33	18.34	10.05	7.57	0.05	0.04	70.67	53.25	–	–	2.03	1.53
LU			166	0.07	0.009	25.88	3.35	30.48	3.95	0.02	0.00	90.24	11.70	–	–	0.09	0.01
LV			331	1.14	0.709	28.21	17.53	28.29	17.57	0.01	0.00	139.86	86.88	–	–	6.60	4.10
NL			935	6.07	0.239	17.98	0.71	204.93	8.07	4.28	0.17	659.44	25.96	0.007	0.00027	6.74	0.27
PL			1456	17.92	6.586	102.76	37.77	165.98	61.01	0.41	0.15	767.53	282.13	0.009	0.0034	9.89	3.64
PT			66	0.04	0.111	3.74	9.76	1.63	4.26	2.34	6.12	10.43	27.23	0.002	0.0049	0.01	0.035
RO			199	0.07	0.031	12.07	5.30	20.16	8.85	0.87	0.38	104.56	45.92	0.007	0.0029	0.73	0.32
SE			1303	1.67	1.289	11.81	9.09	6.31	4.86	32.80	25.26	631.93	486.74	0.004	0.0030	51.36	39.56
SI			106	0.00	0.001	4.42	1.16	2.74	0.72	0.98	0.26	76.01	19.99	0.011	0.0029	0.15	0.040
SK			127	0.03	0.004	7.48	1.17	8.40	1.31	0.41	0.06	92.90	14.52	–	–	0.18	0.028
UK			692	3.25	0.523	7.02	1.13	329.24	53.00	64.42	10.37	114.82	18.48	0.003	0.00056	76.99	12.39

EU			16,127	70	19	781	230	2554	545	253	97	8764	2480	0.7	0.17	243	89.05

**Table:** 

Economic units			*Total*	Ecosystem types													
	Economic sectors	Households		Urban areas		Cropland		Grassland		Heathland and shrub		Woodland and forest		Sparsely vegetated land		Wetlands	
*Year 2012*				NC+	NC	NC+	NC	NC+	NC	NC+	NC	NC+	NC	NC+	NC	NC+	NC
*million euro*																	
AT			955	0.21	0.034	29.84	4.72	141.74	22.40	26.50	4.19	624.02	98.61	0.075	0.0118	2.70	0.43
BE			709	3.75	0.529	143.46	20.25	143.76	20.29	2.33	0.33	323.42	45.66	–	–	4.36	0.62
BG			66	0.02	0.012	4.43	2.15	4.46	2.17	0.29	0.14	34.86	16.96	0.003	0.0013	0.22	0.11
CZ			429	0.23	0.038	35.26	5.75	59.23	9.66	0.28	0.05	272.79	44.48	–	–	1.12	0.18
DE			3728	31.09	6.716	51.75	11.18	749.25	161.86	14.79	3.20	2200.31	475.33	0.018	0.0039	18.83	4.07
DK			22	0.50	0.170	4.17	1.41	0.61	0.21	0.37	0.12	10.28	3.47	–	–	0.56	0.19
EE			40	0.10	0.076	1.70	1.28	1.78	1.34	0.00	0.00	18.28	13.71	–	–	1.08	0.81
EL			39	0.00	0.003	2.05	2.23	1.80	1.96	2.48	2.69	12.11	13.15	0.012	0.0133	0.03	0.03
ES			509	0.15	0.097	30.54	19.66	39.37	25.34	57.21	36.83	181.82	117.04	0.040	0.0259	0.44	0.28
FI			809	0.86	0.339	19.24	7.56	0.19	0.07	3.97	1.56	526.49	206.75	0.001	0.0005	30.28	11.89
FR			2442	0.99	0.190	160.84	30.68	489.69	93.42	29.36	5.60	1360.70	259.57	0.219	0.0417	8.72	1.66
HR			55	0.00	0.005	0.21	9.68	0.06	2.61	0.00	0.12	0.91	41.28	0.0000	0.0002	0.00	0.14
HU			161	0.12	0.022	10.83	2.04	18.63	3.51	0.07	0.01	103.93	19.57	0.002	0.0003	2.22	0.42
IE			156	0.03	0.012	5.98	2.21	74.95	27.67	0.54	0.20	14.73	5.44	0.001	0.0003	17.51	6.46
IT			504	0.12	0.021	32.92	5.88	28.08	5.01	11.82	2.11	353.66	63.13	0.324	0.0578	0.89	0.16
LT			190	1.17	0.875	24.38	18.31	9.65	7.24	0.05	0.04	71.42	53.64	–	–	2.04	1.53
LU			165	0.07	0.009	25.67	3.34	30.16	3.92	0.02	0.00	89.71	11.67	–	-	0.09	0.01
LV			343	1.21	0.739	29.60	18.02	28.69	17.46	0.01	0.00	147.02	89.49	–	–	6.94	4.22
NL			1046	6.70	0.258	20.11	0.78	228.49	8.81	4.79	0.18	739.58	28.51	0.007	0.0003	7.48	0.29
PL			1455	18.24	6.717	102.17	37.64	164.56	60.61	0.40	0.15	768.44	283.05	0.009	0.0034	9.88	3.64
PT			68	0.04	0.120	3.73	10.35	1.62	4.50	2.33	6.47	10.41	28.85	0.002	0.0054	0.01	0.037
RO			199	0.07	0.031	12.04	5.29	20.12	8.83	0.87	0.38	104.52	45.90	0.007	0.0029	0.72	0.32
SE			1301	1.70	1.314	11.76	9.10	6.30	4.88	32.68	25.30	629.60	487.42	0.004	0.0030	51.17	39.61
SI			106	0.00	0.001	4.40	1.16	2.73	0.72	0.98	0.26	75.87	19.95	0.011	0.0029	0.15	0.040
SK			128	0.03	0.004	7.55	1.18	8.56	1.34	0.42	0.07	94.06	14.70	–	–	0.18	0.028
UK			685	3.23	0.517	6.94	1.11	326.09	52.28	63.73	10.22	113.75	18.24	0.004	0.0006	76.33	12.24

EU			16,312	71	19	782	233	2581	548	256	100	8883	2506	0.7	0.18	244	89.42

**Table:** 

Economic units														
	*Total*	Agriculture		Manufacturing & energy production		Construction		Transport		Waste management		Other tertiary and Households		Ecosystem types
*Year 2006*		NC+	NC	NC+	NC	NC+	NC	NC+	NC	NC+	NC	NC+	NC	
*million euro*														
AT	949	18.75	2.79	65.40	10.65	2.42	0.523	73.38	11.71	0.001	0.00013	659.91	103.93	
BE	708	11.04	1.58	89.44	11.72	2.11	0.310	25.57	3.70	0.002	0.00026	492.58	70.31	
BG	67	2.56	1.55	10.50	4.45	0.64	0.677	4.25	2.71	0.000	0.00005	26.80	12.71	
CZ	426	8.18	1.25	80.56	11.63	0.63	0.098	20.81	3.28	0.010	0.00134	256.09	43.29	
DE	3732	148.22	30.51	583.32	117.73	9.41	1.757	209.04	47.50	0.008	0.00134	2120.11	463.95	
DK	22	1.04	0.36	0.42	0.09	0.14	0.035	0.58	0.21	0.000	0.00000	13.91	4.76	
EE	38	2.18	1.54	1.11	1.43	0.48	0.546	3.22	1.89	0.000	0.00000	14.87	10.87	
EL	36	2.85	3.53	1.19	4.12	0.05	0.924	3.01	5.04	0.000	0.00000	11.28	3.77	
ES	478	11.52	14.03	72.36	28.04	9.14	4.478	38.55	40.85	0.005	0.00336	160.29	98.90	
FI	804	10.43	4.86	30.82	15.34	0.00	1.104	45.28	31.33	0.003	0.00119	491.38	173.50	
FR	2432	126.41	25.51	259.70	45.26	4.50	0.647	225.57	56.17	0.008	0.00109	1426.30	262.23	
HR	54	0.59	15.16	0.00	4.99	0.00	0.602	0.31	11.68	0.000	0.00003	0.28	20.48	
HU	156	26.60	5.12	9.98	1.85	1.72	0.283	14.86	2.94	0.002	0.00023	77.80	14.41	
IE	155	15.27	5.95	6.40	1.34	0.98	0.262	11.51	4.58	0.000	0.00001	79.08	29.52	
IT	501	20.26	4.74	86.77	15.24	1.17	0.377	53.05	10.71	0.000	0.00002	264.12	44.56	
LT	190	6.71	4.02	11.65	6.46	0.43	0.349	6.99	3.87	0.001	0.00055	82.50	66.89	
LU	166	1.28	0.16	11.51	1.42	0.00	0.000	15.53	2.01	0.000	0.00000	118.45	15.44	
LV	331	7.12	6.45	24.32	15.30	4.83	2.031	11.77	8.23	0.000	0.00000	156.07	94.78	
NL	935	71.47	2.71	108.38	3.93	84.47	3.235	104.02	3.98	0.002	0.00008	531.11	21.56	
PL	1456	66.45	27.14	76.42	21.89	5.51	1.062	45.52	18.84	0.012	0.00365	870.60	322.35	
PT	66	1.19	4.43	1.91	4.56	0.01	2.429	3.40	11.83	0.000	0.00000	11.68	24.25	
RO	199	12.16	5.22	10.75	5.51	0.29	0.166	7.59	3.71	0.000	0.00008	107.68	46.21	
SE	1303	13.47	8.06	90.11	38.44	0.71	1.062	46.12	66.47	0.000	0.00072	585.47	452.78	
SI	106	3.00	0.95	16.00	5.19	0.14	0.039	14.08	5.33	0.000	0.00000	51.09	10.66	
SK	127	5.19	0.73	16.92	2.50	0.72	0.104	8.32	1.38	0.000	0.00008	78.26	12.38	
UK	692	27.13	4.76	88.51	13.71	2.09	0.318	34.02	6.18	0.003	0.00039	443.99	70.93	

EU	16,127	621	183.1	1754	393	133	23.42	1026	366	0.059	0.015	9132	2495	

**Table:** 

Economic units														
	*Total*	Agriculture		Manufacturing & energy production		Construction		Transport		Waste management		Other tertiary and Households		Ecosystem types
*Year 2012*		NC+	NC	NC+	NC	NC+	NC	NC+	NC	NC+	NC	NC+	NC	
*million euro*														
AT	955	18.65	2.78	67.74	11.07	4.40	0.585	72.96	11.65	0.001	0.0001	661.33	104.30	
BE	709	10.95	1.57	91.51	11.99	2.46	0.391	25.55	3.70	0.002	0.0003	490.61	70.03	
BG	66	2.55	1.54	10.63	4.61	0.02	0.006	4.24	2.70	0.000	0.0001	26.84	12.69	
CZ	429	8.26	1.26	81.68	11.81	0.45	0.098	20.85	3.29	0.009	0.0012	257.66	43.71	
DE	3728	147.12	30.28	593.39	120.76	12.25	2.772	207.03	47.08	0.007	0.0012	2106.25	461.46	
DK	22	1.04	0.36	0.42	0.09	0.00	0.007	0.58	0.21	0.000	0.0000	14.44	4.91	
EE	40	2.18	1.52	1.02	1.32	0.23	0.342	3.26	1.91	0.000	0.0000	16.25	12.12	
EL	39	2.83	3.51	1.26	5.69	0.17	2.120	2.96	5.00	0.000	0.0000	11.26	3.77	
ES	509	11.46	13.94	82.15	37.94	9.09	4.838	37.98	40.22	0.005	0.0041	168.88	102.33	
FI	809	10.39	4.85	30.02	15.28	0.04	1.115	45.34	31.35	0.003	0.0007	495.24	175.58	
FR	2442	125.80	25.39	268.46	46.93	3.77	0.675	223.90	55.70	0.008	0.0011	1428.60	262.47	
HR	55	0.59	15.14	0.00	6.35	0.00	0.233	0.31	11.57	0.000	0.0000	0.28	20.54	
HU	161	26.89	5.14	13.34	2.47	1.55	0.305	15.25	3.06	0.002	0.0002	78.76	14.59	
IE	156	15.28	5.93	6.36	1.33	0.15	0.044	11.53	4.60	0.000	0.0000	80.43	30.08	
IT	504	20.04	4.69	88.22	15.64	3.06	0.870	52.16	10.48	0.000	0.0001	264.32	44.68	
LT	190	6.65	3.99	11.54	6.41	0.70	0.330	6.98	3.87	0.001	0.0005	82.83	67.04	
LU	165	1.27	0.16	9.79	1.27	1.38	0.170	15.46	2.00	0.000	0.0000	117.82	15.36	
LV	343	7.07	6.38	24.32	15.38	2.80	0.752	11.74	8.19	0.000	0.0000	167.53	99.23	
NL	1046	70.23	2.66	138.57	4.74	81.89	3.114	103.03	4.00	0.002	0.0001	613.45	24.31	
PL	1455	66.21	27.05	78.51	22.51	7.74	2.049	45.25	18.79	0.013	0.0037	865.97	321.39	
PT	68	1.17	4.34	1.87	4.51	0.41	5.506	3.33	11.69	0.000	0.0000	11.38	24.27	
RO	199	12.03	5.19	11.20	5.68	0.17	0.117	7.49	3.67	0.000	0.0001	107.44	46.10	
SE	1301	13.45	8.05	90.26	39.74	0.75	0.872	46.24	66.91	0.000	0.0007	582.51	452.06	
SI	106	3.00	0.95	15.95	5.18	0.14	0.039	14.05	5.32	0.000	0.0000	51.03	10.65	
SK	128	5.19	0.73	17.92	2.67	1.18	0.166	8.33	1.39	0.000	0.0001	78.18	12.36	
UK	685	27.05	4.75	86.18	13.12	2.09	0.403	33.87	6.15	0.003	0.0004	440.88	70.18	

EU	16,312	617	182.1	1822	415	137	27.92	1020	364	0.056	0.015	9220	2506	

## Appendix

### Appendix B Derivation of the Curve number values at EU level

We refined the Reference Curve Number (CN) scores (see Section 2.1.1) to assign a different CN to the different CLC classes (${\mathrm{CN}}_{\mathrm{CLC}})$. The refinement was based on the application of the following rules: a) Within each coarse land cover class, assign plus/minus score of 5 depending on the type of vegetation (contrasted with the literature); b) In the case of mixed classes, average values are assigned (i.e. mixed forest); and c) Refined CN scores within each coarse LC class of Table 1 can never reach the values of other coarse LC class. The application of this rules allowed us to estimate the values that appear in the table below black and in bold. The refined values were contrasted with those applied by Hong and Adler (2008) at World level. For this comparison, CN scores of Hong and Adler (2008) were averaged for all soil categories and rescaled between 20 and 77 to match the CN range derived from the correlation coefficients in Table 1. Both approached showed a correlation of 97% (for the black numbers in bold in Table A.1), which confirms the robustness of our approach, but fine-tuned for the EU context. Given the similarity between the CN scores, we used the CN from Hong and Adler (2008) to refine as much as possible those LC classes not included in Schulp et al. 2014a, such as permanent crops and wetlands. The variability of the CN scores across all soil categories was also equivalent to the variability in Hong and Adler (2008).

Table A.1. Processing of the lookup table for the Curve Number scores (CN): from the 'Reference CN' to the 'Refined CN' and the assignment to the different soil categories based on Hong and Adler (2008).^1^Derived from Schulp et al., 2014a (see Table 1).

^2^Land covers excluded: water courses and water bodies, coastal lagoons; beaches, dunes and sands; glaciers and perpetual snow (similarly to Hong and Adler, 2008, Stürck et al., 2014).

^3^Rescaled within the range of the 'Reference CN' (20 and 77).

^4^A. Sand, loamy sand, sandy loam; B. Silt, silt-loam, loam; C. Sandy clay-loam; D. Clay, silty clay, silty clay-loam, sand clay, clay-loam.

^5^Assigned values equivalent to transitional woodland-shrub.

Numbers in bold were used for the comparison of the estimates in this study and curve number scores in Hong and Adler (2008)

### Appendix D Economic assets considered as demand for flood control and correspondence with the economic sectors

^1^Areas covered by rice fields were excluded from the agriculture class as they are already intentionally planted in flooded areas for cultivation.

^2^Based on the Statistical Classification of Economic Activities in the European Community (NACE) to fill in the use table

### Appendix F Accounting tables in monetary terms at country level

*NOTE:* Values at national level for the accounting tables are calculated by summing up the value of the actual ES flow (in biophysical and monetary terms) at sub-catchment level. The allocation of the sub-catchments to the different countries was done based on the position of the sub-catchment centroid. Therefore, transboundary catchments (shared by two countries) were only allocated to the country where the centroid of the sub-catchment is located.

Table A.2. Supply table 2006.Table A.3. Supply table 2012.Table A.3. Use table 2006.Table A.4. Use table 2012.
